# Expression of melanocortin receptors in human endometrium

**DOI:** 10.1093/humrep/dev188

**Published:** 2015-07-29

**Authors:** Anastasia M. Lantang, Barbara A. Innes, Earn H. Gan, Simon H. Pearce, Gendie E. Lash

**Affiliations:** 1 Reproductive and VascularBiology Group, Institute of Cellular Medicine, Newcastle University, Newcastle upon Tyne, UK; 2 Institute of Genetic Medicine, Newcastle University, Newcastle upon Tyne, UK

**Keywords:** melanocortin receptor, endometrium, ACTH, vascular smooth muscle cells, menstrual disorders

## Abstract

**STUDY QUESTION:**

Are melanocortin receptors (MCR1-5) expressed in the endometrium?

**SUMMARY ANSWER:**

MCR1-5 are expressed in endometrium to varying degrees, with MC2R, MC3R and MC5R being the most abundant and the majority of expression being observed in glandular epithelium.

**WHAT IS KNOWN ALREADY:**

Women with Addison's disease who were being administered synthetic ACTH reported menstrual complications as a side effect. There is no previous literature on expression of the melanocortin receptors within the endometrium, and therefore whether ACTH may directly affect the endometrial vasculature.

**STUDY DESIGN, SIZE, DURATION:**

Endometrial biopsies were taken from hysterectomy specimens in control women without endometrial pathology (*n* = 4 for each of proliferative and late-secretory phases). Biopsies were formalin fixed and embedded in paraffin wax. Decidual samples (*n* = 7) were cultured in a range of concentrations of synthetic ACTH for 3 days before being formalin fixed and embedded in paraffin wax.

**PARTICIPANTS/MATERIALS, SETTING, METHODS:**

Endometrial paraffin embedded sections were immunostained for MCR1-5 and assessed using a modified quickscore with luminal epithelium, glandular epithelium, stromal cells, endothelial cells and vascular smooth muscle cells all being assessed separately. Cultured decidual biopsy paraffin embedded sections were immunostained for H-caldesmon and the number of layers of vascular smooth muscle cells surrounding the vessel assessed.

**MAIN RESULTS AND THE ROLE OF CHANCE:**

All five melanocortin receptors were shown to be immunolocalised to the endometrium, with MC5R, MC2R and MC3R being the most abundant and limited immunostaining being observed for MC1R and MC4R. Treatment of decidual biopsies with synthetic adrenocorticotropin (ACTH) resulted in loss of vascular integrity.

**LIMITATIONS, REASONS FOR CAUTION:**

This is an observational study and does not definitively demonstrate a link between synthetic ACTH administration and menstrual complications.

**WIDER IMPLICATIONS OF THE FINDINGS:**

This is the first study to demonstrate widespread expression of melanocortin receptors within the endometrium. Further study is required to determine the role of this hormone family in endometrial function.

**STUDY FUNDING/COMPETING INTEREST(S):**

The work was part funded by MRC grant G09000001. The authors have no competing interests to declare.

**TRIAL REGISTRATION NUMBER:**

Not applicable.

## Introduction

Melanocortin receptors (MCR), constitute a homologous family of five G-protein coupled receptors, MCRs 1 to 5, that mediate the end-organ effects of the signalling peptides encoded by the pro-opiomelanocortin gene, namely the melanocyte stimulating hormones (α to γ MSH), and adrenocorticotropin (ACTH) (Cone, 2006). The system has diverse physiological roles centred around metabolism and energy homeostasis, adrenal steroidogenesis, reproduction, pigmentation, and inflammation (Cone, 2006; Renquist *et al.*, 2011). Their role in the central nervous system (CNS) is the best characterized, with a key role in appetite regulation, satiety and autonomic outflow in CNS tissues. A link between energy homeostasis and reproduction is also mediated by the central effects of leptin signalling, which is permissive to the activity of the hypothalamic gonadotrophin releasing hormone (GnRH) pulse generator, mediated at least in part via MC4R signalling (Ghamari-Langroudi *et al.*, 2011; Xu *et al.*, 2011). This creates the teleologically pleasing phenomenon that reproduction is put on hold by central mechanisms during periods of starvation, caloric restriction and extreme stress. However, a peripheral role for MCR signalling in human reproduction has not been described, although a role for ACTH/MC2R interactions in ovarian steroidogenesis was shown in the pseudo-pregnant rabbit (Guelfi *et al.*, 2011).

During a clinical trial of regular, high-dose tetracosactide (ACTH_1-24_) in patients with autoimmune Addison's disease, we noted that four of nine premenopausal women in the study began to complain of menstrual disturbance (Gan *et al.*, 2014). This took the form of menorrhagia in two women, dysmenorrhoea in one and intermenstrual bleeding associated with uterine pain in one. These side effects of ACTH therapy were well-documented half a century ago when therapeutic use of ACTH was widespread (Treadwell *et al.*, 1964), however, the underlying mechanisms have never been clarified. Endometrial expression of melanocortin receptors would suggest a previously unreported role in female reproductive health. However, it is not known whether this side effect of ACTH administration is due to direct or indirect effects on the endometrium as expression of melanocortin receptors, apart from MC5R, in this tissue has not been previously reported. We therefore examined the expression of the melanocortin receptors 1–5 in human endometrium during different phases of the menstrual cycle.

## Materials and Methods

### Tissue samples

Endometrial biopsies (*n* = 8) were obtained from premenopausal women after hysterectomy for non-endometrial pathology at the Royal Victoria Infirmary, Newcastle upon Tyne. Decidual biopsies (8–10 weeks gestation; *n* = 7) were obtained after termination of apparently normal pregnancies at the Royal Victoria Infirmary, Newcastle upon Tyne. Endometrial biopsies were fixed in 10% (v/v) neutral buffered formalin for 24 h, routinely processed and embedded in paraffin wax. All biopsies included in the study were histologically staged according to standard criteria (Noyes *et al.*, 1975) by a histopathologist and were grouped into late proliferative (*n* = 4) and mid-late secretory (*n* = 4) phases. Decidual biopsies were used fresh for *in vitro* studies. The study was approved by Newcastle and North Tyneside Research Ethics Committee (Ref:10/H0906/71) and all subjects gave written informed consent.

### Immunohistochemistry

Paraffin sections (3 µm) were dewaxed in xylene, rehydrated through alcohols and incubated in 1% (v/v) H_2_O_2_ in methanol for 10 min to block endogenous peroxidase activity. All washes were performed in 0.15M Tris buffered 0.05M saline, pH 7.6 (TBS). All antibodies were fully evaluated on the appropriate control tissue prior to use in the current study. For any given antibody, all tissue sections were immunostained in the same staining run to avoid any day-to-day variation between staining runs.

Antibodies were detected using an avidin biotin peroxidase technique (mouse or rabbit Vectastain Elite ABC kit as appropriate; Vector Laboratories, Peterborough, UK). The immunostaining procedure has been described in detail previously (Schiessl *et al.*, 2009). Details of source, dilution and pretreatment for all primary antibodies are described in Table I. The reaction was developed for 2–3 min with 3, 3′-diaminobenzidine (DAB; Sigma Chemical Co.) containing 0.01% (v/v) H_2_O_2_ to give a brown reaction product. For double labelling (CD56/MC3R and CD56/MC5R) the CD56 was detected using the Vectastain Elite mouse kit and DAB as described above while the second antibody staining was performed with the Vector Immpress AP reagent kit (Vector Laboratories) and detected using the Vector Blue Substrate kit to give a blue reaction product (Vector Laboratories). The sections were lightly counterstained with Mayer's haematoxylin for 30 s, dehydrated, cleared in xylene and mounted with DPX synthetic resin (Raymond A. Lamb Ltd, London, UK). Positive controls were included in each antibody run (Table I). Negative controls were performed for each antibody run and included replacement of the primary antibody by appropriate non-immune serum (goat as supplied in the Vectastain Elite ABC kit). There was no detectable immunostaining in any of the negative control sections and all positive control sections gave the expected immunostaining pattern. All antibodies were evaluated for use with the recommended positive control tissue.

**Table I:** Primary antibodies used in the study.

Antigen	Catalogue number	Species	Dilution	Pretreatment^5^	Positive control
MC1R^1^	Ab125031	Rabbit	1:400	Citrate buffer pH 6.0	Melanoma
MC2R^2^	Sc13107	Rabbit	1:250	EDTA	Adrenal
MC3R^1^	Ab21229	Rabbit	1:50	Trypsin buffer pH 7.8	Brain
MC4R^1^	Ab24233	Rabbit	1:700	Nil	Brain
MC5R^1^	Ab133656	Rabbit	1:100	Trypsin buffer pH 7.8	Brain
H-Cal^3^	M3557	Mouse	1:100	Citrate buffer pH 6.0	Myometrium
CD56^4^	NCL-CD56-1B6	Mouse	1:50	Citrate buffer pH 6.0	Decidua

MCR, melanocortin receptor; H-Cal, H-caldesmon.

^1^AbCam, Cambridge, UK.

^2^Santa Cruz Biotech., Santa Cruz, CA, USA.

^3^Dako, Ely, UK.

^4^Leica Biosystems, Newcastle upon Tyne, UK.

^5^EDTA and citrate buffer pretreatments were pressure cooked for 1 min; trypsin pretreatment was at 37°C for 10 min.

### Analysis of immunostaining

The whole of each immunostained section was analysed semi-quantitatively using a modified ‘Quickscore’ method (Schiessl *et al.*, 2009) taking into account both intensity of staining (0 = negative, 1 = weak, 2 = moderate, 3 = strong) and percentage of cells for each staining intensity (1 = 0–25%, 2 = >25–50%, 3 = >50–75%, 4 = >75–100%). The intensity and percentage scores were then multiplied and summed to give a range of possible scores of 0–12. Luminal epithelium, glandular epithelium, stromal cells, endothelial cells (EC) and vascular smooth muscle cells (VSMC) were all scored separately.

### Decidual biopsy culture

**Figure 1:**
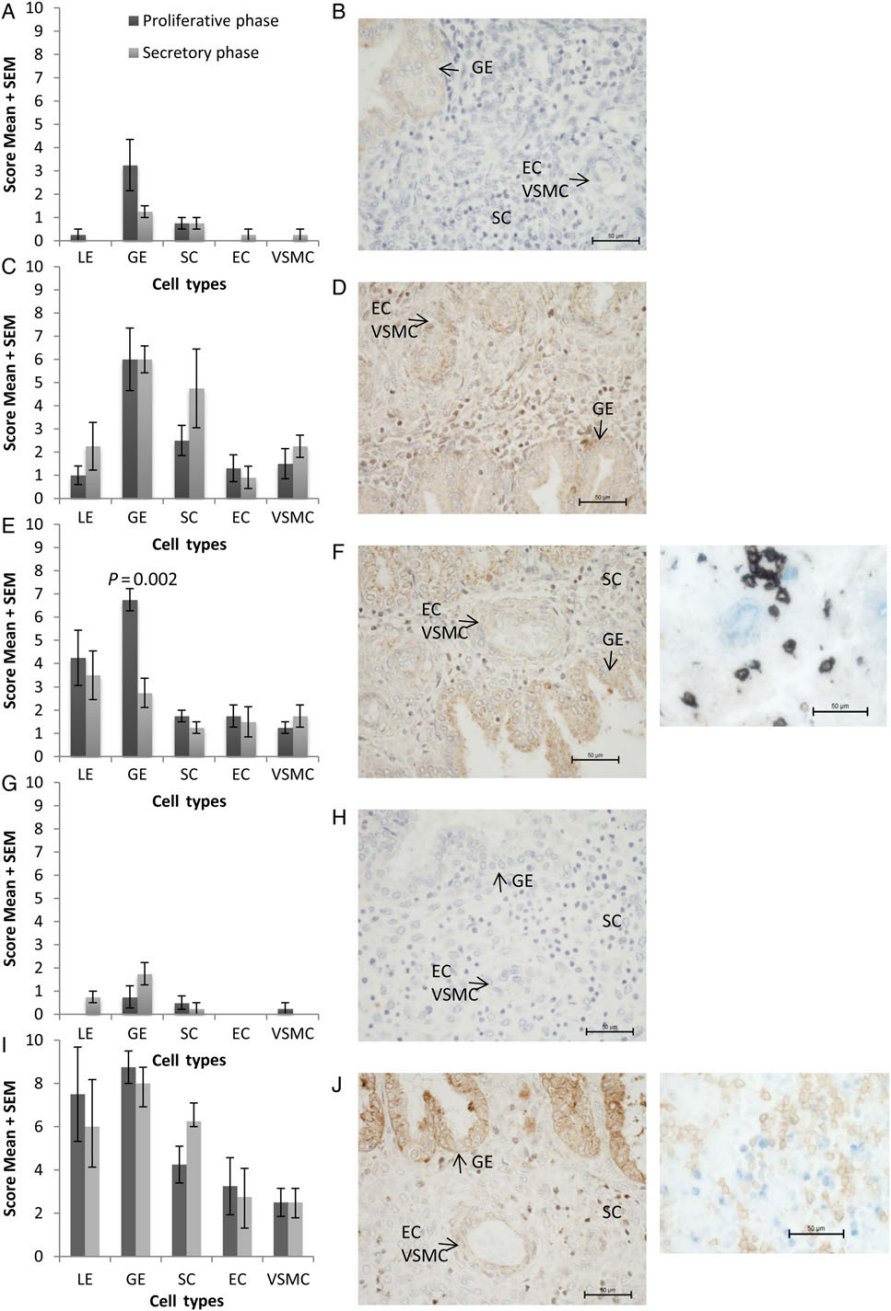
Graphical representation of modified quickscore (mean ± SEM) (**A**, **C**, **E**, **G**, **I**) and representative photomicrograph (**B**, **D**, **F**, **H**, **J**) (original magnification ×400) of endometrial biopsies immunostained for (A, B) melanocortin 1 receptor (MC1R); (C, D) MC2R; (E, F) MC3R; (G, H) MC4R; (I, J) MC5R. (F, H) Insets show double labelling immunohistochemistry for CD56 (brown)/MC3R (blue) (F) and CD56 (brown)/MC5R (blue) (H) (original magnification ×400). LE, luminal epithelium; GE, glandular epithelium; SC, stromal cells; EC, endothelial cells; VSMC, vascular smooth muscle cells. *N* = 4 each group.

**Figure 2:**
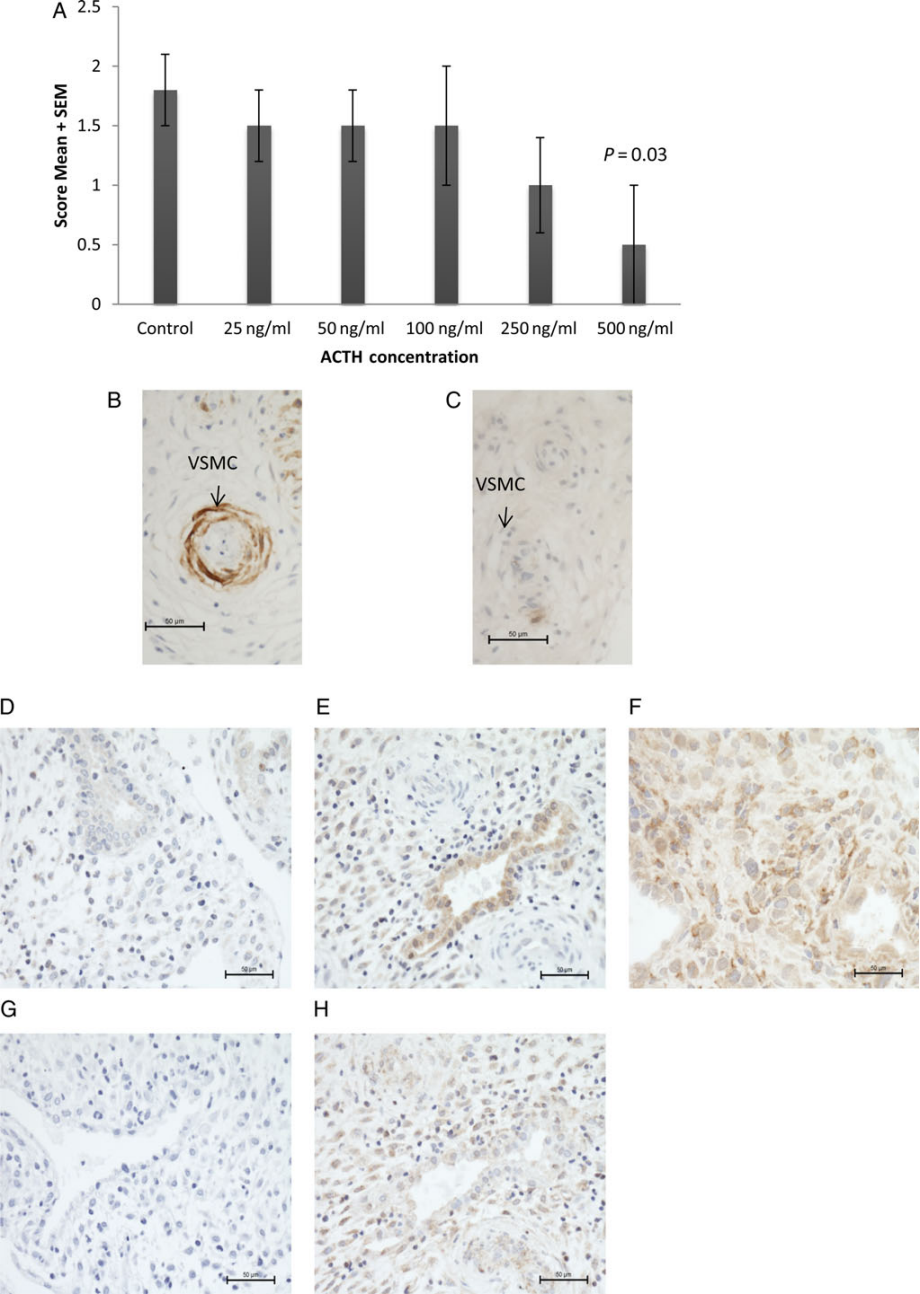
Graphical representation of vascular integrity score (mean ± SEM) (**A**) and representative photomicrographs (**B**, **C**) of decidual biopsies cultured in synthetic adrenocorticotropic hormone (ACTH) for 3 days and immunostained for H-Caldesmon to identify vascular smooth muscle cells. (B) Control sample, (C) after culture in 500 ng/ml synthetic ACTH. *N* = 7 each group. Representative photomicrographs of decidua immunostained for melanocortin 1 receptor (MC1R) (**D**), MC2R (**E**), MC3R (**F**), MC4R (**G**), MC5R (**H**).

Fresh endometrial tissue was not available for culture so decidual biopsies were used. Melanocortin receptor immunostaining patterns were similar in decidua compared with endometrium (compare Figs 1 and 2). Decidual biopsies were washed using PBS, cut into small pieces (∼5 mm^3^) and cultured in a 24 well plate in complete DMEM medium (DMEM medium containing 10% (v/v) calf serum, l-Glutamine, Penicillin/streptomycin and amphotericin B; all from Sigma Chemical Co.). Tissues were treated in different conditions: *T* = 0 (fixed on the day of tissue harvesting), *T* = 3 (cultured in DMEM medium for 3 days), varying concentrations (25, 50, 100, 250 and 500 ng/ml) of ACTH_1-24_ (tetracosactide) diluted in DMEM medium (Alliance Pharmaceuticals Ltd, Chippenham, UK).

The medium was changed every day and tissue harvested after 3 days. The harvested tissue was fixed in 10% (v/v) neutral buffered formalin for 24 h, routinely processed, embedded in paraffin wax and immunostained for H-caldesmon (H-Cal) to identify decidual vessel VSMCs. Vessel integrity was assessed using a modified Quickscore for presence of layers of VSMCs (0 = none, 1 = 1 layer, 2 = 2 or more layers).

### Statistical analysis

Data are presented as means with standard errors. Statistical calculations were performed using the StatView statistical software package (Abacus Concepts Inc., Berkley, CA, USA). Statistical significance was determined by use of ANOVA with *post hoc* Fisher's test or unpaired *t*-test as stated in the text. All statistical tests were two-sided and differences were considered statistically significant at *P* < 0.05.

## Results

### MCR1-5 were immunolocalised to endometrium

#### MC1R

There was weak immunostaining of the glandular epithelium and stroma cells while immunostaining of the luminal epithelium and blood vessels was negligible. There was no statistically significant difference in the staining intensity of MC1R in any of the cell types investigated in the proliferative phase compared with the secretory phase of the menstrual cycle (Fig. 1A and B).

#### MC2R

There was moderate immunostaining of the glandular epithelium, stroma, endothelial cells and vascular smooth muscle cells. There was no marked difference in the staining intensity of MC2R in any of the cell types investigated in the proliferative phase compared with the secretory phase of the menstrual cycle (Fig. 1C and D). Due to antibody incompatibility double labelling with CD56 was not possible. It is interesting to note that immunolocalisation in some of the stromal cells appears to be nuclear, although this may also be reflective of cell size.

#### MC3R

In the proliferative phase of the menstrual cycle there was moderate staining of both the luminal and glandular epithelia with weaker staining in stroma cells and blood vessels. MC3R expression was statistically significantly higher in glandular epithelium of proliferative phase, compared with secretory phase (*P* = 0.002), but otherwise there were no significant differences between proliferative and secretory phases (Fig. 1E and F). Double labelling with CD56, an uterine natural killer cell marker, showed that the majority of MC3R positive stromal cells were uterine natural killer cells.

#### MC4R

There was negligible staining of the glandular epithelium and no significant staining in other structures during either phase of the menstrual cycle (Fig. 1G and H).

#### MC5R

In the proliferative phase of the menstrual cycle there was very strong staining in the luminal and glandular epithelium; moderate staining in stromal cells, endothelial cells and endometrial vascular smooth muscle cell (Fig. 1I and J). High intensity staining was seen in both proliferative and secretory phases. After double labelling with CD56 to identify uterine natural killer cells it was observed that a few of the MC5R positive stromal cells were uterine natural killer cells. Both nuclear and cell surface cellular localization of MC5R could be observed in the stromal cells, including those double labelled with CD56.

### ACTH administration alters vascular integrity

Decidual biopsies were cultured in differing concentrations of ACTH for 3 days prior to immunohistochemical assessment of blood vessel integrity as determined by the number of VSMC layers within the vessel wall. Vascular integrity was reduced, but only after treatment with the highest concentration of ACTH (*P* = 0.03; Fig. 2).

## Discussion

During a clinical trial, we observed menstrual disturbances in premenopausal women as a side effect of medium-term ACTH therapy (Gan *et al.*, 2014). Despite the fact that this has been a well-recognized side effect of ACTH therapy for many years (Treadwell *et al.*, 1964), the molecular underpinning for this phenomenon was not clear. Previously, uterine expression of MC5R mRNA had been reported (Labbé *et al.*, 1994; Chhajlani, 1996), but this had not been examined at the protein level. The expression of other MCRs in endometrium was unknown. In this study, we used immunohistochemistry to survey MCR expression in endometrium, and examined the *in vitro* effects of ACTH stimulation on decidual blood vessels. We confirm robust expression of MC5R in human endometrium at the protein level, and also demonstrate for the first time moderate to strong MC2R and MC3R expression in this tissue. In contrast, MC1R was only very weakly seen in endometrial glandular epithelium, and there was negligible expression of MC4R in any endometrial cell type. As a preliminary functional correlate, we could also observe decreased VSMC content of vessel walls during treatment of decidual tissues with high concentrations of ACTH_1-24_.

Melanocortin 5 receptor is the most widely expressed MCR in mammals, being found in exocrine tissues, thymus and other immune cell types and in certain skeletal muscles (Labbé *et al.*, 1994; Chhajlani, 1996; Van der Kraan *et al.*, 1998). MC5R knock-out mice show a defect in skin sebum secretion (Chen *et al.*, 1997), consistent with the known localization of MC5R in sebaceous glands, and other secretory epithelial structures including rodent lacrimal and preputial glands. Our confirmation of MC5R expression particularly in the luminal and glandular epithelia of the endometrium is consistent with a role for MC5R in secretory epithelia, although there did not appear to be differences in expression between proliferative and secretory phases of the menstrual cycle. This suggests a constitutive role for this receptor, and therefore melanocortin signalling, in endometrium. Moderate MC5R expression on ECs and VSMCs of the endometrial vasculature also suggests that ACTH may have a direct effect on vascular development or remodelling, and therefore contribute to the menstrual disorders observed after administration of synthetic ACTH (Gan *et al.*, 2014).

Melanocortin 3 receptor is believed to be the cognate receptor for γ-MSH, but may also act as a receptor for ACTH and the other MSHs. It is predominantly expressed in the CNS where it acts in concert with MC4R to coordinate feeding rhythms, energy expenditure and homeostasis. MC3R may also have a role in sodium homeostasis (Cone, 2006). While MC3R expression in endometrial epithelial structures was not expected given its currently known central role in energy homeostasis, altered energy intake and food preferences are frequently reported in women during different phases of the menstrual cycle (Buffenstein *et al.*, 1995; Bryant *et al.*, 2006; Hormes and Timko, 2011). These have been assumed to be due to the associated cyclical changes in the steroid hormones, progesterone and estradiol, but the mechanism by which these changes influence feeding behaviours is not clear. It is intriguing to consider whether there may be a melanocortin/ACTH system that centrally regulates feeding behaviour in coordination with the menstrual cycle.

In contrast to MC3R and MC5R, which have wide patterns of expression, MC2R—the cognate ACTH receptor—has a dominant physiological role in adrenal cortex, where it has restricted expression particularly in the zona fasciculata and zona reticularis where it is the key regulator of steroidogenesis and adrenal cell proliferation. Unlike the other melanocortin receptors, MC2R does not act as a receptor for the MSH or the antagonistic agouti peptides. Our study showed robust expression of MC2R in glandular epithelium and to a lesser degree in stromal cells of human endometrium, suggesting a role for ACTH in regulating endometrial glandular secretions.

The endometrium is a dynamic tissue undergoing a regular cycle of growth, shedding and regeneration primarily under the control of the steroid hormones progesterone and estrogen. After menstruation the endometrial stratum functionalis is regenerated from the stratum basalis, which is not shed during menstruation. This regeneration includes repair of the luminal epithelium and re-growth of the endometrial glands, stroma (and associated leucocytes) and vasculature. Development of the endometrial vasculature appears to be key to women's reproductive health, with increased vascular development (increased presence of vascular smooth muscle cells) being associated with recurrent miscarriage and recurrent implantation failure, and decreased vascular development (reduced expression of vascular smooth muscle cell contractile proteins) being associated with heavy menstrual bleeding (Quenby *et al.*, 2009; Biswas Shivhare *et al.*, 2014). In addition, endometrial vascular development has been positively correlated with numbers of uterine natural killer cells; being increased in recurrent miscarriage and recurrent implantation failure and decreased in heavy menstrual bleeding (Quenby *et al.*, 2009; S Biswas Shivhare, JN Bulmer, D Hapangama, GE Lash, personal communication).

Menstrual disturbance is a side effect of synthetic ACTH administration in women, although the mechanism of this disturbance is unclear. In the current study we demonstrate melanocortin receptor expression on both ECs and VSMCs as well as show that high concentrations of ACTH_1-24_ appeared to promote involution of vascular structures in cultured decidua. Therefore ACTH may directly affect ECs or VSMCs leading to vascular instability. Alternatively, ACTH may bind to stromal MCR and elicit alterations in expression of angiogenic growth factors or proteases that in turn act on the vasculature. Uterine natural killer cells are a rich source of angiogenic growth factors that may influence vascular development (Li *et al.*, 2001). We were able to demonstrate uterine natural killer cell expression of both MC3R and MC5R, supporting the idea that ACTH action on vascular stability may be indirect and potentially mediated by uterine natural killer cells surrounding the vessels. However, there was some nuclear localization of melanocortin receptors in the stromal cells, therefore it is unclear whether these receptors are fully functional in this cell type. Further experimentation is required to fully elucidate the mechanism of action of ACTH in altering endometrial vasculature, particularly since the assessment methodology was simplistic and only detected changes at the maximal dose which is likely supraphysiological. However, this could form the basis of the observed side effects seen in the participants of our trial of ACTH therapy. In man, intravenous doses of 2–5 µg of ACTH_1-24_ (tetracosactide, synacthen) are known to produce maximal adrenal cortisol responses, and in our clinical trial 1 mg bolus doses of synacthen depot were administered on alternate days, giving rise to the possibility of supraphysiological effects been found in our participants. However, we were not able to measure tissue levels of ACTH_1-24_ in these women and therefore tested a wide range of concentrations in our *in vitro* assay system.

In conclusion, this study forms a descriptive foundation on which to base further investigations of the effect of ACTH and melanocortins on endometrial function: a subject which we now clearly show to have some clinical relevance. It may be speculated that the melanocortin receptors mediate non-pregnant endometrial, and pregnant decidual, steroidogenesis, vascular stability and have functions central to human reproductive health.

## Authors' roles

A.M.L. was involved in study execution, data collection and analysis. B.A.I. was involved in sample collection and preparation. E.H.G. was involved in study design and contributed towards critical discussion of the manuscript. S.H.P. and G.E.L. played roles in study design, data analysis and wrote the manuscript.

## Funding

The work was part funded by MRC grant G09000001. Funding to pay the Open Access publication charges for this article was provided by Newcastle University.

## Conflict of interest

None declared.
